# Unbiased Subgenome Evolution in Allotetraploid Species of *Ephedra* and Its Implications for the Evolution of Large Genomes in Gymnosperms

**DOI:** 10.1093/gbe/evaa236

**Published:** 2020-11-16

**Authors:** Hui Wu, Qiong Yu, Jin-Hua Ran, Xiao-Quan Wang

**Affiliations:** 1 State Key Laboratory of Systematic and Evolutionary Botany, Institute of Botany, Chinese Academy of Sciences, Beijing, China; 2 University of Chinese Academy of Sciences, Beijing, China

**Keywords:** transcriptome, genome evolution, unbiased subgenome evolution, allotetraploid, *Ephedra*, gymnosperm

## Abstract

The evolutionary dynamics of polyploid genomes and consequences of polyploidy have been studied extensively in angiosperms but very rarely in gymnosperms. The gymnospermous genus *Ephedra* is characterized by a high frequency of polyploidy, and thus provides an ideal system to investigate the evolutionary mode of allopolyploid genomes and test whether subgenome dominance has occurred in gymnosperms. Here, we sequenced transcriptomes of two allotetraploid species of *Ephedra* and their putative diploid progenitors, identified expressed homeologs, and analyzed alternative splicing and homeolog expression based on PacBio Iso-Seq and Illumina RNA-seq data. We found that the two subgenomes of the allotetraploids had similar numbers of expressed homeologs, similar percentages of homeologs with dominant expression, and approximately equal numbers of isoforms with alternative splicing, showing an unbiased subgenome evolution as in a few polyploid angiosperms, with a divergence of the two subgenomes at ∼8 Ma. In addition, the nuclear DNA content of the allotetraploid species is almost equal to the sum of two putative progenitors, suggesting limited genome restructuring after allotetraploid speciation. The allopolyploid species of *Ephedra* might have undergone slow diploidization, and the unbiased subgenome evolution implies that the formation of large genomes in gymnosperms could be attributed to even and slow fractionation following polyploidization.

**Significance**

The evolutionary dynamics of polyploid genomes and consequences of polyploidy have been very rarely studied in gymnosperms. Our previous study showed that the gymnospermous genus *Ephedra* is characterized by a high frequency of allotetraploidy, and thus provides an ideal system to explore the evolutionary mode of allopolyploid genomes and investigate whether subgenome dominance has occurred in gymnosperms. Our present study investigated the subgenome evolution in two allotetraploid species of *Ephedra* and found that, unlike most allopolyploid species of angiosperms, the two *Ephedra* species showed an unbiased subgenome evolution, indicating a slow diploidization. This finding also implies that the formation of large genomes in gymnosperms could be attributed to even and slow fractionation following polyploidization.

## Introduction

A high frequency of polyploidy or whole-genome duplication (WGD) plays an important role in plant evolution and has broad effects on phenotypic diversification, ecological tolerance, and species richness for both autopolyploids and allopolyploids (Otto 2007; Paterson et al. 2010; Fawcett et al. 2013; Weiss-Schneeweiss et al. 2013; Jiao and Paterson 2014; Wendel 2015; Soltis PS and Soltis DE 2016; Rice et al. 2019). Following WGD, generally, the polyploids will revert to a stable status, similar to diploids, by fractionation, the loss of one copy of duplicated genes or *cis*-regulatory sites (Wendel 2015; Dodsworth et al. 2016; Soltis PS and Soltis DE 2016). In addition, subgenome dominance is commonly associated with polyploid evolution, with the dominant subgenome showing genome-wide high expression levels and more alternative splicing events (Liu et al. 2014; Mei et al. 2017) and retaining more ancestral genes (i.e., biased fractionation) immediately and over the long term (Cheng et al. 2018; Bird et al. 2019; Edger et al. 2019). Subgenome dominance has been observed in many allopolyploids of varying ages, such as *Mimulus peregrinus* (140 years old, Edger et al. 2017), *Arabidopsis suecica* (0.02 Ma, Novikova et al. 2017), maize (8 Ma, Schnable et al. 2011), *Brassica rapa* (15 Ma, Wang et al. 2011), *Arabidopsis thaliana* (47 Ma, Thomas et al. 2006), and *Medicago sativa* (58 Ma, Garsmeur et al. 2014), whereas it does not occur in autopolyploids such as *Populus trichocarpa* (Liu et al. 2017) and *Musa acuminata* (Garsmeur et al. 2014), and even a few allopolyploids such as soybean (*Glycine max*) (Zhao et al. 2017), *Cucurbita maxima*, and *Cucurbita moschata* (Sun et al. 2017).

In contrast to the high frequency of polyploids documented in angiosperms, polyploidy is exceedingly rare in gymnosperms, although gymnosperms are characterized by large genome sizes, with a mean value of 1 C = 18.35 pg, which is much larger than that of angiosperms (1 C = 5.9 pg) (Leitch and Leitch 2013). Nevertheless, recent studies have suggested that the evolution of gymnosperms was accompanied by several ancient WGD events (e.g., Li et al. 2015; Guan et al. 2016; Roodt et al. 2017; One Thousand Plant Transcriptomes Initiative 2019), and polyploidy is a dominant mode of speciation in *Ephedra*, a unique genus with 83% of the species being polyploids or having polyploid cytotypes (Wu et al. 2016; Ickert-Bond et al. 2020). Compared with numerous studies of the diploidization process in angiosperm allopolyploids, few studies have investigated the evolutionary dynamics and consequences of polyploidy in gymnosperms (only *Juniperus* in Farhat et al. 2019, and *Ephedra* in Ickert-Bond et al. 2015, 2020). Studies of the origin, accumulation, and fate of duplicated functional genes are helpful to unravel the mechanisms underlying genome evolution, including the evolution of large genome size and important pathways in gymnosperms. Moreover, it has been hypothesized that gymnosperms might not have an efficient way to eliminate nonfunctional gene copies, as observed in the accumulation of long terminal repeat retrotransposons (LTR-RTs), leading to the accumulation of degenerate gene copies and gene-like sequences (Prunier et al. 2016). However, this hypothesis needs to be tested empirically.

*Ephedra* provides an ideal system for investigating evolutionary dynamics of polyploid genomes and consequences of polyploidy in gymnosperms. With the exception of the natural polyploid species in three genera of Cupressaceae (*Fitzroya cuprssoides* and *Sequioa sempervirens*, Ahuja 2005; 17.3% of *Juniperus* species, Farhat et al. 2019) and sporadic polyploids with multiple and aneuploid chromosome numbers in *Amentotaxus* (Chuang and Hu 1963; Guan et al. 1993; Zhou et al. 2000), *Pseudolarix amabilis* (Murray 2012), *Encephalartos hildebrandtii* (Abraham and Mathew 1966), and *Gnetum montanum* (Ickert-Bond and Renner 2016), all other natural polyploid species of gymnosperms belong to *Ephedra*, in which 83% of species show tetraploid or very rarely octoploid cytotypes (Khoshoo 1959; Huang et al. 2005; Wu et al. 2016; Ickert-Bond et al. 2020). In particular, these polyploid species originated mainly in the Neogene with a crown age of all extant *Ephedra* species dated to ∼30 Ma (Ickert-Bond et al. 2009), although the earliest fossil record of the genus was dated to the Early Cretaceous (Yang and Wang 2013). Based on the analyses of two single-copy nuclear genes (*LFY* and *DDB*2) and two chloroplast DNA fragments, Wu et al. (2016) inferred that all polyploid species of *Ephedra* distributed in the Qinghai–Tibetan Plateau (QTP) and neighboring areas, such as *Ephedra saxatilis*, *Ephedra intermedia*, and *Ephedra sinica*, are allotetraploids, and deduced that the high frequency of allopolyploid speciation could be associated with some biological features of *Ephedra*, such as a shrub habit and vegetative propagation. The allotetraploid *E. sinica* is a shrub or small erect herbaceous shrub that tends to be clonal, with a vast distribution from northwestern China northward to Mongolia and Russia and eastward to the Gulf of Bohai. *Ephedra sinica* has been used as a traditional Chinese medicine for over 5,000 years and is still being used in various *Ephedra*-containing herbal mixtures all over the world (Hagel et al. 2012). This species experienced a WGD event, by tetraploidization after hybridization with one diploid species most closely related to *Ephedra przewalskii* and *Ephedra regeliana* as the maternal donor and another diploid species most closely related to *Ephedra equisetina*–*Ephedra minuta*–*Ephedra monosperma* as the paternal donor (Wu et al. 2016). The allotetraploid *E. intermedia* also has a vast distribution and its putative progenitors are similar to those of *E. sinica*. Moreover, significant ecological divergence has occurred between the allotetraploid species and their putative progenitors (Wu et al. 2016). Therefore, it is of great interest to investigate how the subgenomes of these allopolyploid species evolved and whether the expression patterns and evolutionary dynamics of their subgenomes are correlated to the genome size evolution and biological features.

Although RNA-seq using short-read sequencing technology has been increasingly used in studying plant transcriptomes, it is still challenging to assemble transcriptomes of allopolyploid species without reference genome sequences because homeologs are difficult to disentangle, particularly when the divergence between subgenomes is low at the sequence level. In contrast, using long-read sequencing technology, full-length isoforms can be directly obtained from sequencing without assembly (Sharon et al. 2013). Recently, Pacific Biosciences (Pacific Biosciences of California Inc., Menlo Park, CA) single-molecule real-time long-read isoform sequencing has performed well in sequencing transcriptomes of cotton, maize, and sorghum, especially accurately predicting alternative splicing and revealing transcriptomic complexity (Abdel-Ghany et al. 2016; Wang et al. 2016, 2018). In the present study, we selected *Ephedra sinica*, *E. intermedia*, and their putative progenitors to investigate subgenome evolution in allotetraploid species of *Ephedra*. First, isoforms of tetraploids were generated on PacBio Iso-Seq, and were used to identify expressed homeologs and explore the patterns of alternative splicing. Then, transcriptome sequencing was conducted on the Illumina HiSeq 2000/2500 platform, and was used to investigate homeolog expression of the two subgenomes. Finally, based on a comprehensive analysis of genome size, expression patterns of subgenomes, and biological attributes, we discussed the mechanisms underlying the evolution of large genomes and the possible correlation between allopolyploid speciation and some biological features in gymnosperms.

## Materials and Methods

### Plant Sampling and an Outline of Methods

Two allotetraploid species (*Ephedra sinica* and *E. intermedia*) and their putative diploid progenitors (*E. equisetina*, *E. minuta*, *E. monosperma*, *E. przewalskii*, and *E. regeliana*), and an outgroup species *Ephedra rhytidosperma* (based on our unpublished research) were sampled. For the two allotetraploid species, a total of 8 samples, representing different tissues (young stems and female strobili) and environments (field and green house), were analyzed. For the five diploid species and the outgroup, each species was represented by only one sample (young stem) collected in the field. All 14 samples were immediately immersed in RNAlater Solution (Life Technologies) after collection. The details of sampling are shown in table 1 and supplementary table S1, Supplementary Material online.

**Table 1:** Statistics of Sampled Transcriptomes

Category	Species	Ploidy Level	Pop.	Sample	Illumina Sequencing and Data Analyses		PacBio Iso-Seq and Data Analyses		
					No. of Reads After Filtering	No. of CDS	No. of Polymerase Reads	No. of Consensus Transcripts	No. of High-Quality Consensus Transcripts
Putative maternal progenitors	*Ephedra equisetina*	2*x*	ZL	S	30,714,049 (100 bp)	30,158			
	*Ephedra monosperma*	2*x*	YX	S	37,104,728 (100 bp)	27,102			
	*Ephedra minuta*	2*x*	MY	S	29,722,252 (100 bp)	34,623			
Putative paternal progenitors	*Ephedra przewalskii*	2*x*	KLMY	S	51,820,340 (100 bp)	28,992			
	*Ephedra regeliana*	2*x*	YS	S	30,074,428 (100 bp)	26,883			
Polyploids	*Ephedra sinica*	4*x*	XW	S	21,987,457 (100 bp)		1,129,502	179,191	34,517
				F	23,947,858 (100 bp)		1,035,706	184,925	38,655
			KB	S	20,206,814 (150 bp)		929,043	159,591	26,623
				F	18,600,852 (150 bp)		764,474	184,929	46,439
			XL-W	S	18,902,491 (150 bp)		586,803	123,351	26,370
				F	18,724,916 (150 bp)		566,519	131,275	28,437
			XL-C	S	20,526,994 (150 bp)		688,763	152,536	29,753
	*Ephedra intermedia*	4*x*	INT	S	15,570,339 (150 bp)		481,241	98,103	21,950
Outgroup	*Ephedra rhytidosperma*	2*x*	RHY	S	32,633,573 (100 bp)	32,414			

Note.—XL-C, transplanted from population XL and cultivated in the green house of the Institute of Botany, Chinese Academy of Sciences.

Combining the advantages of Illumina sequencing and PacBio Iso-Seq, our study was conducted as follows: 1) full-length transcriptomes of polyploid samples were sequenced by PacBio Iso-Seq, and transcriptome sequences of diploid samples were obtained from de novo assembly of short reads generated on the Illumina platform; 2) expressed homeolog identification and alternative splicing analysis were performed for the full-length transcriptomes of polyploid samples based on the reference of one-to-one orthologous groups (OGs) identified from transcriptomes of diploid species; 3) single-nucleotide polymorphisms (SNP) analysis was further conducted based on the reads of both diploids and polyploids generated on the Illumina platform to investigate homeolog expression patterns of the two subgenomes of polyploids with the transcriptome of *E. regeliana* (a putative diploid progenitor) as the reference, due to the lack of a reference genome in *Ephedra* and the complexity of transcriptomes of polyploid species (supplementary fig. S1, Supplementary Material online).

### Illumina Sequencing and Data Analyses

For all samples, total RNA was extracted using the RNAplant Plus Reagent (Tiangen, China). Sequencing libraries were prepared using a NEBNext Ultra RNA Library Prep Kit for Illumina (NEB) and then sequenced on an Illumina HiSeq 2000/X-Ten platform with 100-bp/150-bp paired-end raw reads (supplementary table S1, Supplementary Material online). For the five diploid samples, reads filtering and de novo assembly were performed with Trimmomatic 0.38 (Bolger et al. 2014), Trinity 2.0.6 (Grabherr et al. 2011; Haas et al. 2013), CD-HIT 4.6.8 (Li and Godzik 2006), and TransDecoder 0.36 (https://github.com/TransDecoder, last accessed December 08, 2020), as described in Ran et al. (2018). The completeness of transcripts was evaluated using BUSCO (Benchmarking Universal Single-Copy Orthologs) v4 (Simão et al. 2015) with the Embryophyta (odb10) database. One-to-one OGs were identified by OrthoFinder 2.1.2 (Emms and Kelly 2015), following Liu et al. (2019).

### PacBio Iso-Seq and Data Analyses

For the polyploid species, cDNA of each sample was synthesized using the SMARTer PCR cDNA Synthesis Kit (Clontech Laboratories, CA). PCR amplification and size fractionation (1–6 kb, 0.5–6 kb) were conducted using the KAPA HiFi PCR Kits (Kapa Biosystems) and BluePippin Size Selection System (Sage Science), respectively. Libraries were constructed using the SMRTbell Template Prep Kit 1.0 (PacBio) and sequenced on a PacBio Sequel Platform. Sequence data were analyzed using SMRT Link 5.1 (http://www.pacb.com/products-and-services/analytical-software/smrt-analysis/, last accessed December 08, 2020). The raw reads were filtered with the settings of length >100, pass >3, and accuracy >0.75, and then selected nonchimeric reads were classified into nonfull-length reads and full-length reads, which were determined by a length of at least 300 bp and presence of poly(A) tails, 5′ primers and 3′ primers. Further, full-length reads were processed by isoform-level clustering (ICE) to obtain unpolished consensus transcripts. Finally, full-length consensus transcripts were polished using the Quiver software module for error correction and categorized into high-quality consensus transcripts (min_accuracy >0.99, min_pass >2) and low-quality consensus transcripts. The high-quality consensus transcripts were corrected based on the corresponding Illumina RNA-seq data using the software Proovread 2.14.0 (Hackl et al. 2014), and then the coding sequences (CDS) were predicted using TransDecoder 0.36 (https://github.com/TransDecoder, last accessed December 08, 2020), and redundant sequences were removed using CD-HIT 4.6.8 (Li and Godzik 2006). The completeness of high-quality consensus transcripts was evaluated by using BUSCO4 (Benchmarking Universal Single-Copy Orthologs) (Simão et al. 2015) with the Embryophyta (odb10) database.

### Identification of Expressed Homeologs

For the allopolyploid samples, the subgenomes derived from the paternal parent and the maternal parents were designated as P subgenome and M subgenome, respectively. Because genic regions of the P and M subgenomes were highly similar, we developed a pipeline to separate these homeologs based on homeologous SNPs matched, respectively, to the sequences of the putative paternal parents *E. equisetina*–*E. minuta*–*E. monosperma* and the maternal parents *E. przewalskii*–*E. regeliana* in the alignment of one-to-one OGs using custom MATLAB 2014b scripts snp_based_on_sequence.m (https://github.com/yazhicao/Ephedraanalysis/, last accessed December 08, 2020). Consensus transcripts were aligned to the OGs identified in the five diploid species using BLAST. We removed low quality or short sequences from the alignment and counted the number of SNPs in each consensus transcript that are only shared with one parent, corresponding to maternal (M) sites and paternal (P) sites. Based on the SNPs, after removing recombination sequences, the sequences with at least four M sites were considered as homeologs from subgenome M, and the sequences with at least four P sites were considered as homeologs from subgenome P. We set a minimum of 4 SNPs because of the low site variation in the coding sequences. Only the isoforms that can be unambiguously assigned to the parental species were included in the analysis. The isoforms with the proportion of M sites >0.8 were assigned to subgenome M, and those with the proportion of M sites <0.2 were assigned to subgenome P.

To verify the accuracy of the above pipeline, a phylogenetic approach was also used to identify expressed homeologs. The alignments of the OGs identified in the five diploid species and the orthologous consensus transcripts of polyploid samples were, respectively, used to construct ML trees using RaxML 8.2.11 (Stamatakis 2014) with 100 bootstrap replicates and the GTRGAMMA model. After excluding the trees with bootstrap support values lower than 60% at node *E. equisetina*–*E. minuta*–*E. monosperma*-consensus transcript of polyploids and node *E. przewalskii–E. regeliana*-consensus transcript of polyploids, statistics of expressed homeologs were performed using Newick utilities 1.7.0 (Junier and Zdobnov 2010).

Gene ontology (GO) annotation of the identified OGs was obtained by Blast2GO program (Conesa et al. 2005) against the Nr annotation, and GO categories were analyzed using the Web Gene Ontology Annotation Plot (WEGO 2.0) (Ye et al. 2018).

To investigate the expression patterns of homeologs, pairwise Pearson correlations were examined between samples and a clustering map was drawn based on the expressed homeologs of each OG. Moreover, to explore the minimal number of consensus transcripts that are required to obtain the maximum number of expressed OGs and OGs that homeolog pairs from both parents are expressed, a series of subdata sets, including 30,000–150,000 sequences with an increment of 30,000 sequences with 3 replicates, were extracted from the consensus transcripts of the samples KB-F and XW-S, respectively. For each subdata set, the identification of expressed homeologs was performed as mentioned earlier. Then, we used the polynomial function of degree 2 in MATLAB 2014b (poly_curve.m) to fit the saturation curve between the logarithmic number of consensus transcripts and the logarithmic number of expressed OGs, and between the logarithmic number of consensus transcripts and the logarithmic number of OGs where both homeologs are expressed, respectively. The two polynomial functions are expressed as:

$$\text{log}\;\left(y_{1}\right)=-0.177{\left(\text{log}\;\left(x\right)\right)}^{2}+1.69\;\text{log}\;\left(x\right)+4.7385,$$

$$\text{log}\;\left(y_{2}\right)=-0.1013{\left(\text{log}\;\left(x\right)\right)}^{2}+0.7389\;\;\text{log}\;\left(x\right)+7.2712,$$

where *x* is the number of consensus transcripts, *y*_1_ is the number of expressed OGs, and *y*_2_ is the number of OGs.

### Phylogenetic Analysis and Divergence Time Estimation

Phylogenetic relationships of the polyploids and their putative diploid progenitors were reconstructed using the OGs found in the diploids that have homeologs (high-quality consensus transcripts) in both subgenomes of the polyploids, with *E. rhytidosperma* as the outgroup. The different samples of polyploids were used, separately, in the identification of homeologs, and then the homologous OGs found in different tissue samples of the same individual were combined in the phylogenetic analysis. For the OGs with multiple high-quality consensus transcripts of the polyploid samples, only one transcript with the maximum length and the least numbers of single-nucleotide insertions/deletions was retained from each subgenome, and then the errors of single-nucleotide insertions/deletions were manually corrected.

Both concatenation and coalescence strategies were used in phylogenetic reconstruction. In the concatenation analysis, all OGs were combined into a concatenated supermatrix using FASConCAT-G 1.02 (Kück and Longo 2014), and a maximum-likelihood (ML) tree was generated by RaxML 8.2.11 (Stamatakis 2014) using the GTRGAMMA model with 100 bootstrap replicates. In the coalescence analysis, the ML tree was generated for each OG using RaxML 8.2.11 with the same parameter settings as above, and then all individual gene trees were used to estimate the species tree in ASTRAL 5.7.3 (Mirarab et al. 2014).

To estimate the divergence times between the putative diploid progenitors and the ages of the polyploids, the OGs of one individual of *E. sinica* (KB) and *E. intermedia* were used to determine density distributions of synonymous substitution rates (*Ks*), considering that the two subgenomes of *E. sinica* form reciprocal monophyletic groups (see Results). We estimated *Ks* for sequence pairs using paraAT 2.0 (Zhang et al. 2012). After excluding *Ks* values <0.001 to avoid spurious frequency peaks, Gaussian mixture models were used to identify significant peaks in the *Ks* distribution with the best fitting model selected based on Bayesian information criterion scores using script gaussian_analysis.m. According to the phylotranscriptomic study of gymnosperms (Ran et al. 2018), an average mutation rate of 4.8 × 10^−9^ synonymous substitutions per synonymous site per year for *Ephedra* was used to estimate approximate ages of the polyploids.

### Analysis of Alternative Splicing

Alternative splicing (AS) analysis can also provide evidence for gene expression patterns in different subgenomes. Recent studies have shown that it is feasible to use PacBio sequences to identify AS events by searching for deletions or insertions in the clustering units when reference genomes are unavailable (Ner-Gaon et al. 2007; Zhou et al. 2011; Wu et al. 2014; Liu et al. 2017). Based on the alignments composed of consensus transcripts from all samples of *E. sinica* or *E. intermedia* for each one-to-one OG, the longest CDS of the allotetraploids was predicted by the merge of high-quality consensus isoforms’ CDS and validated as the reference. The AS events of each OG were identified with lengths of deletion or insertion >51 bp from the alignment.

### SNP Analysis

Due to the lack of a reference genome in *Ephedra*, filtered clean reads of both diploid and polyploid samples were mapped to *E. regeliana* (the best reference species based on the phylogenetic analysis, see Results) using BWA-MEM (Li and Durbin 2009), with default parameters. The mapped reads were sorted with SAMtools 1.1 (Li et al. 2009). Variant calling was conducted using HaplotypeCaller and GenotypeGVCFs in Genome Analysis Toolkit GATK 3.6 (McKenna et al. 2010; DePristo et al. 2011). To obtain high-quality SNPs, variant sites were filtered using GATK’s VariantFiltration tool based on the following criteria: quality of depth <2.0, Fisher strand bias (FS) >10.0, mapping quality (MQ) <40.0, depth of coverage (DP) <30.0, ReadPosRankSum <−8.0, and genotype quality (GQ) <20.0. We focused on SNPs that are not shared between two parents, and then classified them into heterozygous sites (S_PM_) that have fixed differences between two subgenomes for polyploid samples_,_ or homozygous sites (S_PP_ or S_MM_) that are only shared with maternal or paternal parents, where PP and MM represent paternal and maternal homozygosities, respectively (SNP_analysis.m). The autapomorphic SNPs in either the diploids or one subgenome of the polyploids were excluded from the analysis. Further, genes with at least four informative “heterozygous” sites or four homozygous sites were classified into G_PM_, G_PP_, or G_MM_. For these genes, homeolog-specific expression was measured by calculating the proportion of all reads mapping to the subgenome P using the DepthPerAlleleBySample values found in the VCF file.

### Genome Size Estimated by Flow Cytometry

Fresh young branchlets were used in the flow cytometry measurement for each species, mainly following the one-step protocol of Doležel et al. (2007). *Vicia faba* L. “Inovec” (2 C = 26.90 pg) (Doležel et al. 1992) was selected as the internal standard and Galbraith’s buffer was used as the most appropriate nuclei isolation buffer. The DNA ploidy levels were inferred based on the DNA content measured in *E. equisetina* (2 C = 16.61 pg), a diploid species with 14 chromosomes (Wu et al. 2016).

## Results

### Transcriptome Data Collection and Processing

A total of 8 transcriptomes, representing different tissues (young stems and female strobili) and environments (field and green house), were generated from the two allotetraploid species *E. sinica* and *E. intermedia* using PacBio Sequel and Illumina platforms. For the full-length transcriptomes, we obtained 16.7–30 G raw data for each sample, including 481,241–1,129,502 polymerase reads with average lengths of 14,752–43,461 bp. The mean number of passes in polymerase reads was almost higher than 15, indicating that the circular consensus sequences have a high accuracy according to the PacBio sequencing study of Eid *et al.* (2009) (15 passes may yield >99% accuracy). After the clustering step, a total of 98,103–184,929 consensus transcripts were collected and the average lengths were 1,350–2,257 bp, of which 21,950–46,439 were high-quality transcripts (min_accuracy >0.99, min_pass >2) (table 1 and supplementary table S2 and fig. S2, Supplementary Material online). For the Illumina HiSeq data, an average of 42.94 million (M) clean reads were obtained per sample (table 1).

In addition, transcriptome sequences of the five putative progenitors *E. przewalskii*, *E. regeliana*, *E. equisetina*, *E. minuta*, and *E. monosperma* were generated on the Illumina platform and used as references. For each sample, we obtained 41,602–69,343 transcripts with an N50 value of 1,464–1,703 bp from de novo assembly. The number of predicted CDS varied from 26,883 to 34,623 (table 1 and supplementary table S1, Supplementary Material online), from which 6,245 one-to-one OGs were obtained, with aligned length ranging from 150 to 6,735 bp. Based on the BUSCO assessment, the completeness of the transcripts from diploids is better than that of the high-quality transcripts from polyploids (supplementary fig. S3, Supplementary Material online).

### Characterization of Expressed Homeologs Based on PacBio Iso-Seq Data

The consensus transcripts of 8 polyploid samples were aligned to the 6,245 one-to-one OGs. The expressed homeologs corresponding to the P subgenome (represented by *E. equisetina*–*E. minuta*–*E. monosperma*) and the M subgenome (represented by *E. przewalskii*–*E. regeliana*) were identified based on the homeologous SNPs. The OGs with expressed homeolog pairs from both subgenomes were denoted as H_MP_, and the OGs with expressed homeologs only from subgenome M or subgenome P were denoted as H_M_ or H_P_. After filtering, 206,314 isoforms (accounting for 47% of the aligned isoforms and 17% of all isoforms, supplementary table S3, Supplementary Material online) were well-classified into 5,402 OGs, with the mean length of isoforms ranging from 974.8 to 1,311.1 bp and the average SNPs per isoform ranging from 13.1 to 17.3. The numbers of transcripts assigned to subgenome M and subgenome P, and the proportions of M sites in the isoforms are shown in supplementary figure S4, Supplementary Material online. Among the 5,402 OGs, 4,893 were annotated to Nr annotation and classified into 3 groups based on GO terms, including 3,523 in “biological process,” 4,773 in “cellular component,” and 3,845 in “molecular function” (supplementary fig. S5, Supplementary Material online).

In *E. sinica*, we obtained 3,438–4,389 expressed OGs from different samples, with the average number of isoforms ranging from 5.1 to 10.8 (supplementary table S4, Supplementary Material online). For each sample, the H_MP_ expression was detected in most OGs (2,164–3,285, accounting for 62–75%). In contrast, the H_M_ expression and the H_P_ expression occurred in fewer OGs and in approximately equal proportions. Similar homeolog expression patterns were found in *E. intermedia*, in which H_MP_, H_M_, and H_P_ in identified 3,097 OGs accounted for 60%, 19%, and 21%, respectively (table 2). Among all of the samples, the female strobili of an individual of *E. sinica* from population KB (KB-F) showed the highest number of expressed OGs (4,389), the most consensus transcripts (184,929) with the highest average number of isoforms per OG (10.8), and the highest H_MP_ expression (75%) (table 2 and supplementary table S4, Supplementary Material online). These results indicated that no obvious expression difference occurred between the two subgenomes (M and P) of the allotetraploid species, although this analysis was only based on the presence/absence of expressed homeologs and the expression patterns showed a little difference between tissues. The unbiased homeolog expression in different subgenomes was also revealed by the phylogenetic analysis (supplementary table S5, Supplementary Material online).

**Table 2:** Distribution Patterns of Expressed Homeologs

SampleCategory	*Ephedra sinica*									*Ephedra intermedia*	
	XW			KB			XL-W			XL-C	INT
	S	F	S + F	S	F	S + F	S	F	S + F	S	S
Total	3,509	3,995	4,528	3,614	4,389	4,789	3,438	3,575	4,369	3,514	3,097
H_MP_	2,164 (62%)	2,636 (66%)	3,364 (74%)	2,571 (71%)	3,285 (75%)	3,833 (80%)	2,257 (65%)	2,481 (69%)	3,364 (77%)	2,492 (71%)	1,874 (60%)
H_M_	614 (17%)	641 (16%)	535 (12%)	480 (13%)	504 (11%)	423 (9%)	575 (17%)	534 (15%)	475 (11%)	470 (13%)	576 (19%)
H_P_	731 (21%)	718 (18%)	629 (14%)	563 (16%)	600 (14%)	533 (11%)	606 (18%)	560 (16%)	530 (12%)	552 (16%)	647 (21%)

Note.—H_MP_, OGs with expressed homeolog pairs from both subgenomes; H_M_, OGs with expressed homeologs only from subgenome M; H_P_, OGs with expressed homeologs only from subgenome P; S, young stems; F, female strobili.

**Fig. 1:**
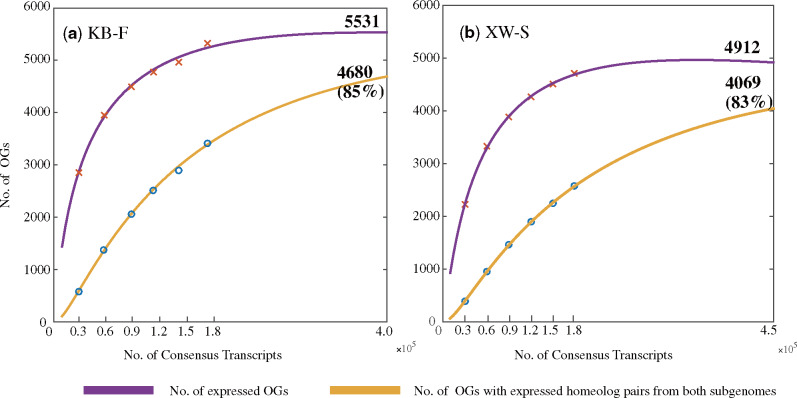
Saturation curves used to predict the maximum numbers of expressed OGs and OGs with expressed homeolog pairs from both subgenomes.

Based on the expression patterns of homeologs, no obvious correlation was found between the polyploid samples, with the pairwise Pearson correlation coefficients ranging from 0.20 to 0.42 (supplementary table S6, Supplementary Material online). The distributions of the expressed homeologs in each OG are shown in supplementary figure S6, Supplementary Material online, which also does not show obvious correlation between samples except that 825 OGs with expressed homeolog pairs from both subgenomes are shared among at least seven samples. In addition, H_M_ and H_P_ mostly (60–81%) occurred in OGs at a low coverage with one to four isoforms (supplementary table S4, Supplementary Material online). To further investigate the maximum number and proportion of expressed OGs and H_MP_, the saturation curve was used to predict the relationship between the number of consensus transcripts and the number of expressed OGs, and between the number of consensus transcripts and the number of H_MP_. We found that, in the sample KB-F, the saturation values of expressed OGs and H_MP_ were close to 5,531 and 4,690, respectively, indicating that the proportion of H_MP_ could be close to 85% when the number of consensus transcripts ≥400,000. In the young stem sample of an individual of *E. sinica* from population XW (XW-S), the proportion of H_MP_ was also up to 83% with 450,000 consensus transcripts (fig. 1).

### Phylogenetic Reconstruction and Divergence Time Estimation

Among the 6,245 OGs found in the diploid species, 3,953 OGs matched homeologs (high-quality consensus transcripts) in the two subgenomes of the two polyploid species, including 2,627 OGs in XW, 2,802 OGs in KB, 2,231 OGs in XL-W, and 1,033 OGs in XL-C of *E*. *sinica*, and 871 OGs in INT of *E*. *intermedia*, which were used to infer phylogenetic relationships. The length of the concatenated sequences was 4,666,169 bp, with 179,269 variable sites and 86,671 parsimony-informative sites.

**Fig. 2:**
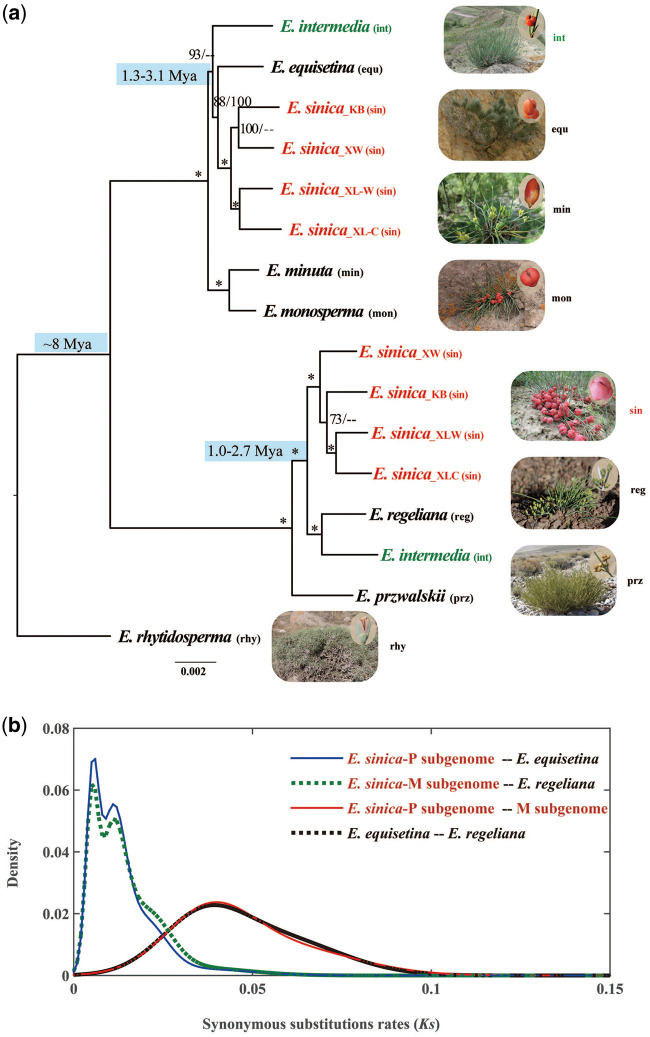
Phylogenetic analysis based on the concatenation method and divergence time estimation. (*a*) A maximum-likelihood tree showing the phylogenetic relationships among the two subgenomes of two polyploid species and their putative diploid parents. Numbers associated with nodes are bootstrap support (BS) values obtained from the concatenation (left) and coalescent (right) analysis, respectively. An asterisk indicates BS of 100%. Diploids are in black, and polyploids are in color. (*b*) Density distribution of *Ks* among the two subgenomes of *Ephedra sinica* and its putative diploid parents.

The phylogenies reconstructed based on the concatenation and coalescence methods are largely consistent in topology with high bootstrap support (fig. 2*a*). Two clades were resolved, one containing *E. equisetina*, *E. minuta*, *E. monosperma*, *E. sinica*-P subgenome, and *E. intermedia*-P subgenome, and the other comprising *E. przewalskii*, *E. regeliana*, *E. sinica*-M subgenome, and *E. intermedia*-M subgenome. In particular, the P subgenomes of the two polyploid species formed one monophyletic subclade with the diploid *E. equisetina*, and the M subgenomes of them formed another monophyletic subclade (100% bootstrap support) with the diploid *E. regeliana*. This result suggested that the two polyploid species very likely originated from hybridization with the two diploid species as parents, although the possibility of *E. monosperma*/*E. minuta* as the paternal progenitor of *E. intermedia* cannot be ruled out given the low bootstrap support for a close relationship between *E. intermedia* and *E. equisetina* in the coalescent tree.

Based on the *Ks* analysis, we estimated the divergence times between *E. equisetina* and *E. regeliana* (putative diploid parents), between the two subgenomes of the polyploids, and between each subgenome and the corresponding paternal/maternal progenitor. The density distributions of *Ks* values are shown in figure 2*b* and supplementary figure S7, Supplementary Material online, and the Gaussian components are listed in supplementary table S7, Supplementary Material online. The mixture model analysis indicated the presence of peaks at 0.04 for both between the two subgenomes of *E. sinica* and *E. intermedia* and between *E. equisetina* and *E. regeliana*, and the divergence times between them were estimated to be ∼8 Ma. However, the divergence between each subgenome and the corresponding paternal/maternal progenitor occurred much later, at 1.0∼3.1 Ma (supplementary table S7, Supplementary Material online).

### Identification of Alternative Splicing without a Reference Genome

One of the most important features of Iso-Seq is to give access to the direct detection of AS by directly comparing isoforms of the same gene. Based on the clustering isoforms in the 5,204 OGs, we carefully analyzed AS in *Ephedra*. The reference of each OG was predicted by the merge of high-quality consensus isoforms’ CDS, with an average length of 1,343 bp for *E. sinica* and 1,292 bp for *E. intermedia*. For the polyploid samples of *E. sinica* and *E. intermedia*, we detected 337–1,343 AS events from 314 to 1,179 isoforms, which occurred in 267–857 OGs, accounting for 8.62–21.45% of all identified OGs. The numbers of isoforms with AS from subgenome M and from subgenome P were approximately equal for all samples (table 3).

**Table 3:** Characterization of Alternative Splicing (AS) Events in *Ephedra sinica* and *Ephedra intermedia*

No.	Sample	*Ephedra sinica*							*Ephedra intermedia*
		XW		KB		XL-W		XL-C	INT
		S	F	S	F	S	F	S	S
Subgenome M	Isoforms (%)	457 (2.08%)	576 (2.03%)	411 (1.61%)	553 (1.46%)	208 (0.93%)	358 (1.44%)	450 (1.65%)	160 (0.89%)
	AS events	534	658	450	604	220	387	510	174
	OGs (%)	389 (11.09%)	489 (12.24%)	363 (10.04%)	464 (10.57%)	187 (5.44%)	312 (8.73%)	379 (10.79%)	140 (4.52%)
Subgenome P	Isoforms (%)	433 (1.97%)	603 (2.12%)	465 (1.83%)	565 (1.49%)	228 (1.02%)	351 (1.41%)	446 (1.64%)	154 (0.86%)
	AS events	484	685	500	620	254	373	499	163
	OGs (%)	365 (10.4%)	508 (12.72%)	392 (10.85%)	475 (10.82%)	208 (6.05%)	315 (8.81%)	390 (11.10%)	143 (4.62%)
All	Isoforms	890	1,179	876	1,118	436	709	896	314
	AS events	1,018	1,343	950	1,224	474	760	1,009	337
	OGs (%)	644 (18.35%)	857 (21.45%)	659 (18.23%)	803 (18.30%)	341 (10.00%)	555 (15.52%)	668 (19.00%)	267 (8.62%)
All isoforms		22,017	28,392	25,454	37,892	22,445	24,906	27,224	17,984
Identified OGs		3,509	3,995	3,614	4,389	3,438	3,575	3,514	3,097

### Homeolog Expression Based on SNP Analysis

**Fig. 3:**
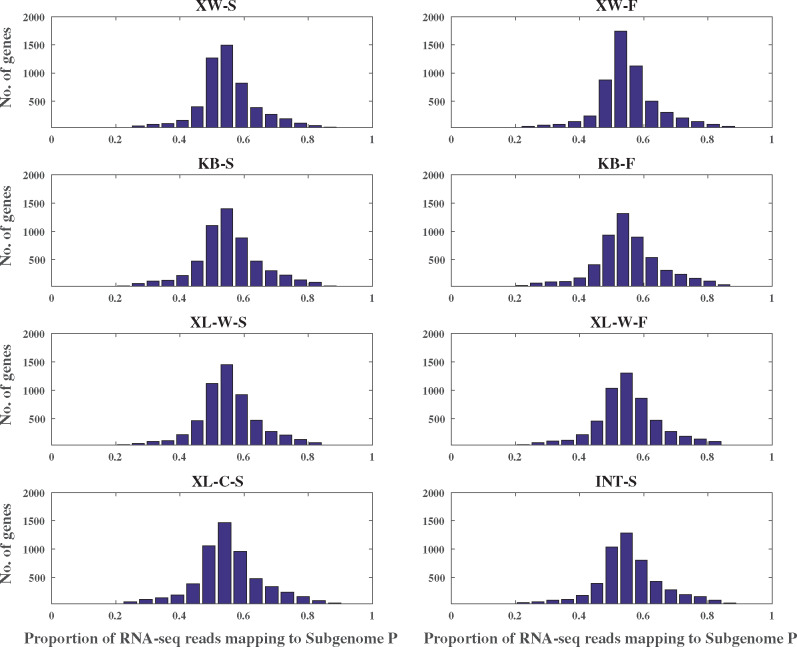
Transcriptome analyses for homeolog expression.

Based on the RNA-seq data, we identified a total of 159,933 SNPs which differed between the putative paternal parent *E. equisetina* and maternal parent *E. regeliana*, representing the differences between subgenome P and subgenome M. Of these SNPs, the polyploid samples’ sites were annotated with respect to variants. The most abundant SNPs were S_PM_ sites (80,351–94,151 per sample, accounting for 76–79%). The S_PP_ and S_MM_ sites were far less abundant (S_PP_: 12,219–14,855 per sample; S_MM_: 10,721–14,642 per sample). Corresponding to the types of SNPs, 5,279–5,835 G_PM_, 298–358 G_PP_, and 265–537 G_MM_ genes were identified per sample. The gene distributions showed that ∼90% of genes in the expressed transcripts had expressed homeolog pairs from both subgenomes across all samples (table 4). If the percentage of an expressed homeolog is higher than 0.6, we defined it as the dominant homeolog. The percentage of homeologs with dominant expression in subgenome M was close to the percentage of homeologs with dominant expression in subgenome P in different samples. A large number of genes (78–83%) showed conserved expression levels (fig. 3). Thus, there appeared to be no differences between subgenomes in the number of expressed genes or the overall expression patterns.

**Table 4:** Distributions of SNPs and Genes in Polyploid Samples

Sample	Category		No. of SNPs			No. of Genes		
			S_PM_	S_PP_	S_MM_	G_PM_	G_PP_	G_MM_
*Ephedra sinica*	XW	S	87,443	13,856	14,179	5,488	341	509
		F	90,253	14,117	14,642	5,672	342	537
	KB	S	91,812	14,417	13,313	5,823	358	453
		F	87,696	13,862	13,261	5,634	354	479
	XL**-**W	S	86,696	13,529	14,156	5,725	325	538
		F	81,541	12,834	12,602	5,455	303	456
	XL**-**C	S	94,151	14,855	10,721	5,835	363	265
*Ephedra intermedia*	INT	S	80,351	12,219	13,456	5,279	298	452

Note.—S_PM_, heterozygous sites that have fixed differences between two subgenomes; S_PP_ or S_MM_, sites that only shared with one subgenome; G_PM_, G_PP_, and G_MM_, genes with at least four sites.

### Genome Size Diversity

Based on the flow cytometry measurement, the genome size of the putative maternal progenitor *E. regeliana* was 14.86 pg, and that of the putative paternal progenitor *E. equisetina* was slightly larger. The nuclear DNA contents of the allotetraploids *E. sinica* and *E. intermedia* were almost equal to the sum of two putative progenitors (table 5), consistent with the study of Ickert-Bond et al. (2020).

**Table 5:** Genome Sizes of the *Ephedra* Species Estimated by Flow Cytometry

Species	Holoploid Genome Size (1C-value, pg)	SD	CV%	Inferred Ploidy Level	Monoploid Genome Size (1Cx-value, pg)
*Ephedra minuta*	8.19	0.02	3.15	2*x*	8.19
*Ephedra equisetina*	8.30	0.01	2.63	2*x*	8.30
*Ephedra monosperma*	8.54	0.01	1.85	2*x*	8.54
*Ephedra regeliana*	7.43	0.03	2.01	2*x*	7.43
*Ephedra przewalskii*	7.65	0.01	3.53	2*x*	7.65
*Ephedra sinica*	15.42	0.03	2.46	4*x*	7.71
*Ephedra intermedia*	16.06	0.04	1.98	4*x*	8.03

Note.—SD, standard deviation; CV, calculated coefficient of variation.

## Discussion

### Unbiased Subgenome Evolution in Allotetraploid Species of *Ephedra*

In the past two decades, numerous studies have yielded valuable insights into the evolutionary dynamics of polyploid genomes and consequences of polyploidy in angiosperms, but very rarely in gymnosperms (Bird et al. 2018). Previous studies also indicate that subgenome dominance is often associated with allopolyploid evolution, although unbiased WGD has been reported in a few allopolyploids (Cheng et al. 2018; Liang and Schnable 2018). However, based on the analyses of transcriptome sequences generated from PacBio Iso-Seq and Illumina HiSeq, our present study found unbiased subgenome evolution in two allotetraploid species of *Ephedra*, a unique gymnosperm genus with 83% of the studied 52 species being polyploids (Ickert-Bond et al. 2020). The two allotetraploid species *E. sinica* and *E. intermedia* possibly originated from hybridization with *E. regeliana* as the maternal parent and *E. equisetina* as the paternal parent with the divergence time of two subgenomes at 8 Ma, although the paternal progenitor of *E. intermedia* has not been completely resolved (fig. 2).

This finding is supported by several lines of evidence. First, genes of the putative diploid progenitors are retained in the two subgenomes of the allotetraploids in similar numbers. Expressed homeolog pairs from both subgenomes (H_MP_) were detected in 60–75% OGs, and the H_MP_ expression can reach 85% as predicted by the saturation curve (fig. 1). In the remaining OGs, the H_M_ expression is also approximately equal to the H_P_ expression in percentage (table 2 and supplementary fig. S6, Supplementary Material online). In addition, the numbers of isoforms with alternative splicing are approximately equal between subgenome M and subgenome P for all samples (table 3). Moreover, the SNP analysis indicates that 76–79% of surveyed sites are heterozygous with fixed differences between two subgenomes and ∼90% of genes show expressed homeolog pairs from both subgenomes across all samples (table 4). These results are similar to the observations in a few allopolyploids of angiosperms such as *G. max* (Garsmeur et al. 2014), *C. maxima* and *C. moschata* (Sun et al. 2017), and *Pyrus bretschneideri* (Li et al. 2019), which have two ancestral subgenomes with similar gene numbers and show unbiased fractionation. Second, expression dominance was not found between two subgenomes. The percentage of homeologs with dominant expression in subgenome M is close to that of homeologs with dominant expression in subgenome P, and large numbers of genes (78–83%) generally show conserved expression levels (fig. 3). These results strongly contrast with the reports from most allopolyploids of angiosperms such as maize (Swigonová et al. 2004; Schnable et al. 2011) and *B. rapa* (Wang et al. 2011; Cheng et al. 2016), which show expression dominance and many more events of alternative splicing in one subgenome (Liu et al. 2014; Mei et al. 2017). Finally, the nuclear DNA content of the allotetraploid species is almost equal to the sum of two putative progenitors, suggesting limited genome restructuring after allotetraploid speciation (table 5), as reported in Ickert-Bond et al. (2015, 2020). This characteristic is similar to the modes in *G. max* (Garsmeur et al. 2014), *Capsella bursa-pastoris* (Douglas et al. 2015), the allotetraploid *Cucurbita* species (Sun et al. 2017), and *Eragrostis tef* (VanBuren et al. 2020), which display karyotype stability after polyploidization.

### Implications of the Unbiased Subgenome Evolution for the Formation of Large Genomes in Gymnosperms

For most allopolyploid species of angiosperms, a large fraction of genes from progenitor genomes were lost in the subsequent diploidization process, showing biased fractionation (Soltis PS and Soltis DE 2016; Van de Peer et al. 2017a, 2017b). Subgenome expression dominance is one important mechanism responsible for biased fractionation (Yoo et al. 2014; Cheng et al. 2018). Mechanistically, the unequal gene expression between duplicates may result in differential fitness, leading to biased gene loss with respect to ancestral genomes (Freeling et al. 2012; Bottani et al. 2018; Cheng et al. 2018; Wendel et al. 2018). For example, studies on maize genomes showed that the homeologs in the overfractionated subgenome tend to have lower levels of gene expression, relaxed selection, and higher gene loss, but this evolutionary pattern did not occur in soybean (Pophaly and Tellier 2015; Renny-Byfield et al. 2017; Zhao et al. 2017). In maize, the estimated 85% of originally duplicate gene pairs have become reduced singletons, and the chromosomes (2*n* = 20) are almost equal to its diploid outgroups sorghum (2*n* = 20) and rice (2*n* = 24) (Schnable et al. 2011; Brohammer et al. 2018). In contrast, although the soybean experienced a tetraploidization event (13 Ma, Schmutz et al. 2010) at roughly the same time as maize (11.4 Ma, Gaut and Doebley 1997), soybunderwent slow diploidization, retaining the majority of duplicates and containing 40 chromosomes (2*n* = 40) that are nearly double the number of chromosomes in the common bean (2*n* = 22) and pigeon pea (2*n* = 22) (Du et al. 2012; Zhao et al. 2017).

The unbiased subgenome evolution found in the allotetraploid species of *Ephedra* leads us to infer that these polyploids might also have undergone slow diploidization with limited genome downsizing. This inference is also supported by previous studies on two conifer genera *Sequoia* and *Juniperus* (Scott et al. 2016; Farhat et al. 2019). The unbiased subgenome evolution might also have contributed to the formation of large genomes in gymnosperms considering that at least one round of WGD occurred before the divergence of seed plants (Jiao et al. 2011) and several ancient WGD events occurred in the evolution of gymnosperms (e.g., Li et al. 2015; Guan et al. 2016; Roodt et al. 2017), although some of these WGD events remain controversial (Zwaenepoel and Van de Peer 2019). The accumulation of transposable elements, accounting for 74%, 76.58%, 79%, and 85.9% of the genomes of *Pinus taeda* (Neale et al. 2014; Wegrzyn et al. 2014), *Ginkgo biloba* (Guan et al. 2016), *Pinus lambertiana* (Stevens et al. 2016), and *Gnetum montanum* (Wan et al. 2018), respectively, could be attributed to slow rates of chromosome rearrangements, as evidenced not only by the cytological stability between diploid and polyploid species in *Ephedra* (this study, Ickert-Bond et al. 2020), possibly with the formation of disomic inheritance similar to wheat (Yousafzai et al. 2010; Mercier et al. 2015) but also by the moderate genome downsizing following polyploidization in *Juniperus* (Farhat et al. 2019), and a high degree of synteny between *Picea* and *Pinus* (Pavy et al. 2012). In addition, in the large genome of gymnosperms, there is a surprisingly large fraction of gene-like sequences or pseudogenes, in which gene-like sequences represent 2.4% and 2.9% of the *Picea abies* and *Pinus taeda* genomes, respectively (Nystedt et al. 2013; Neale et al. 2014). Moreover, a large fraction of gene duplications predated the angiosperm–gymnosperm split. The unbiased subgenome evolution of the allotetraploid species of *Ephedra* further supports the hypothesis that gymnosperms might lack a mechanism for eliminating redundant gene copies (Nystedt et al. 2013; Warren et al. 2015).

Notably, in *Ephedra*, 83% of species show tetraploid or very rarely octoploid cytotypes (Ickert-Bond et al. 2020), with a high frequency of allopolyploid speciation reported in Asia (Wu et al. 2016). Why has a high frequency of polyploidy occurred in *Ephedra*? From the biological view, clonal reproduction is positively associated with polyploidy incidence in angiosperms (Husband et al. 2013; Weiss-Schneeweiss et al. 2013; Freeling 2017; Van Drunen and Husband 2019). The high percentage of polyploid species in *Ephedra* could be related to a shrub habit and clonal propagation. In contrast to the large trees in conifers, all species of *Ephedra* are perennial shrubs or sometimes vines or small trees, and have underground rhizomes. The rhizomes of *Ephedra* can sometimes reach as long as several meters (Pearson 1929), even in rocky slopes (our field investigation), which provide a good mechanism for vegetative propagation (Cutler 1939) and could be helpful to the survival of polyploids. The vegetative propagation also partially contributed to the success of the hexaploid *Sequoia sempervirens* (Scott et al. 2016) and polyploid *Juniperus* species (e.g., in *Juniperus sabina* and *Juniperuscommunis*; Houle and Babeux 1994; Wesche et al. 2005). The complete genome sequencing of *Ephedra* species and comparative genomic analyses will further reveal the mechanisms underlying the genome evolution, speciation, and adaptation of the genus.

## Supplementary Material

evaa236_Supplementary_Data

## Data Availability

The sequence data are deposited in GenBank under the BioProject accession PRJNA602052. The sequence alignments are deposited at Dryad under the accession: doi: 10.5061/dryad.bvq83bk5g.
