# Supplementary material for: Unbiased Subgenome Evolution in Allotetraploid Species of Ephedra and Its Implications for the Evolution of Large Genomes in Gymnosperms
Source: Genome Biol Evol. 2020 Nov 16;13(2):evaa236. doi: 10.1093/gbe/evaa236 (PMC7900875; doi:10.1093/gbe/evaa236)
Supplement: evaa236_Supplementary_Data [file evaa236_supplementary_data.docx]

**The following Supporting Information is available for this article:**

**Fig. S1** Pipeline for data analysis

**Fig. S2** Length distribution of consensus transcripts

**Fig. S3** Results of the BUSCO assessment

**Fig. S4** Proportion of M sites per transcript from subgenome M (a) and from subgenome P (b)

**Fig. S5** GO functional classification of the OGs. The x-axis represents the GO annotation categories and the y-axis represents the percentage of OGs of a specific category/total matched OGs

**Fig. S6** Distributions of expressed homeologs of each OG in each polyploid sample. Each row corresponds to a sample and each column corresponds to an OG

**Fig. S7** Density distribution of *Ks* among two subgenomes of *E. intermedia* and its putative diploid parents. Ei-P: subgenome P of *E. intermedia*; Ei-M: subgenome M of *E. intermedia*; equ: *E. equisetina*; reg: *E. regeliana*.

**Table S1** Information of the transcriptome data generated from Illumina HiSeq

**Table S2** Information of the transcriptome data generated from PacBio Iso-Seq

**Table S3** Information of analyzed transcripts

**Table S4** Summary of isoform distribution

**Table S5** Distribution patterns of expressed homeologs based on phylogenetic approach

**Table S6** Pearson’s correlation coefficients between polyploid samples based on expression patterns of homeologs

**Table S7** Complete mixture model estimates of *Ks* distributions


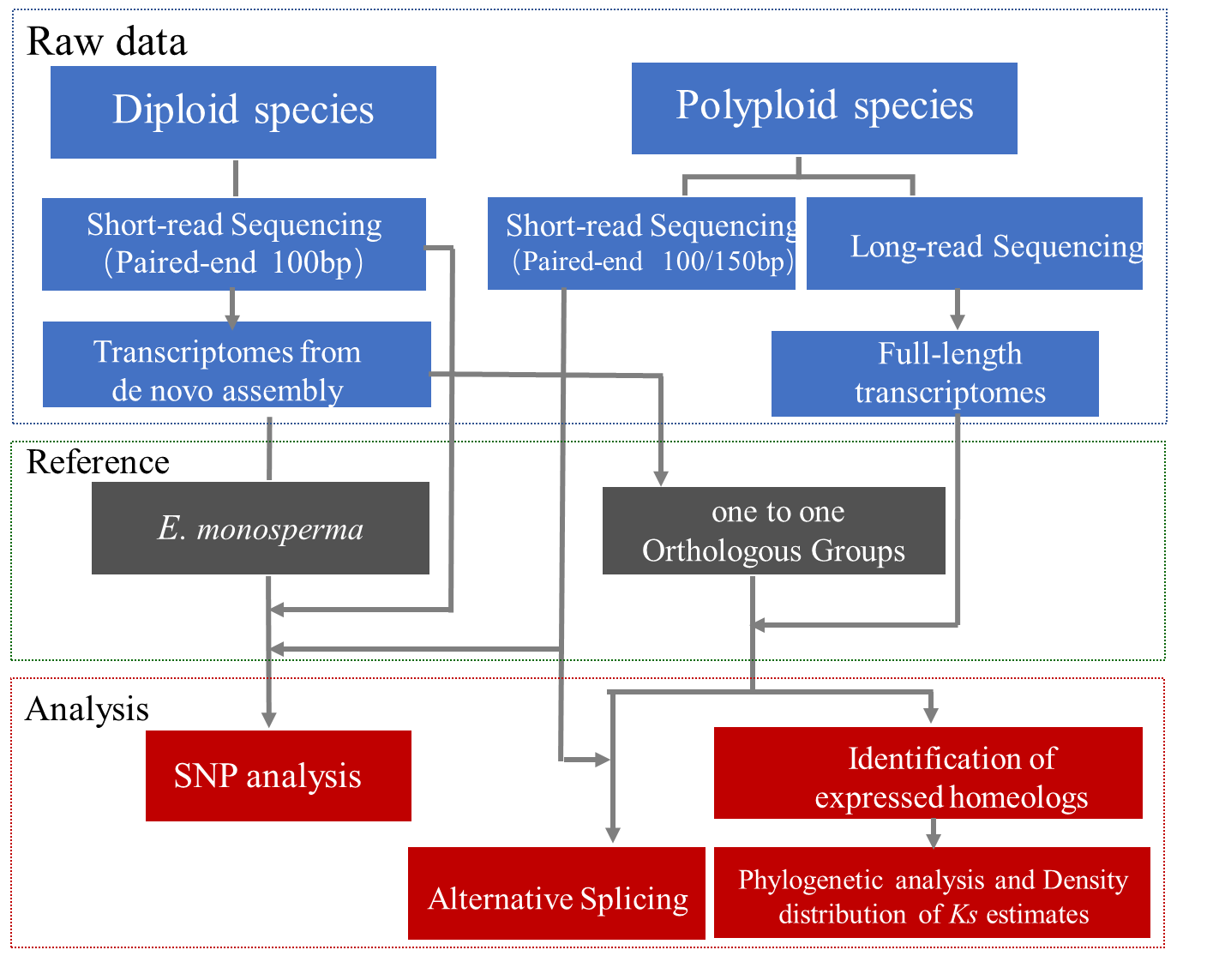


**Fig. S1 Pipeline for data analysis**


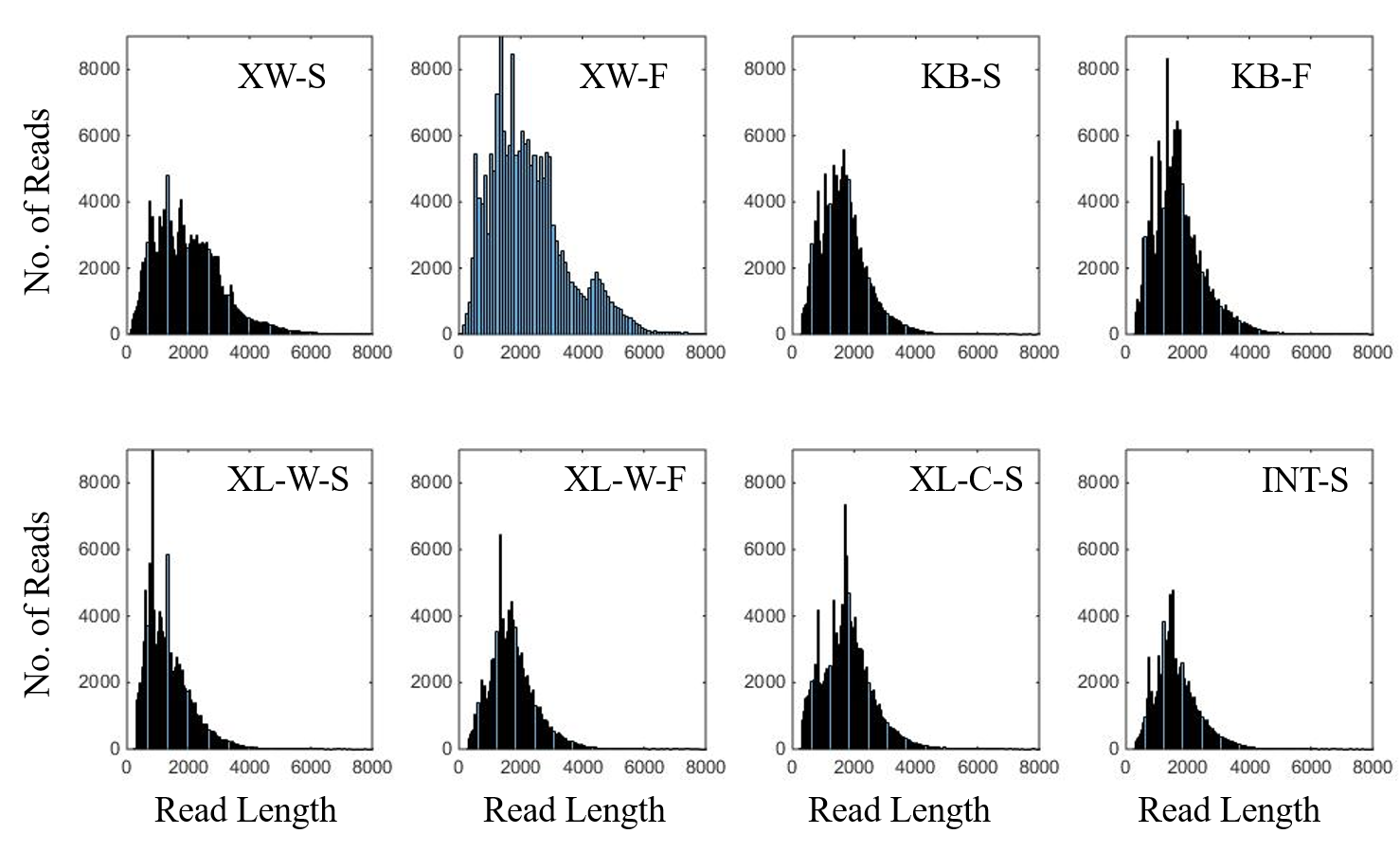


**Fig. S2 Length distribution of consensus transcripts**

**
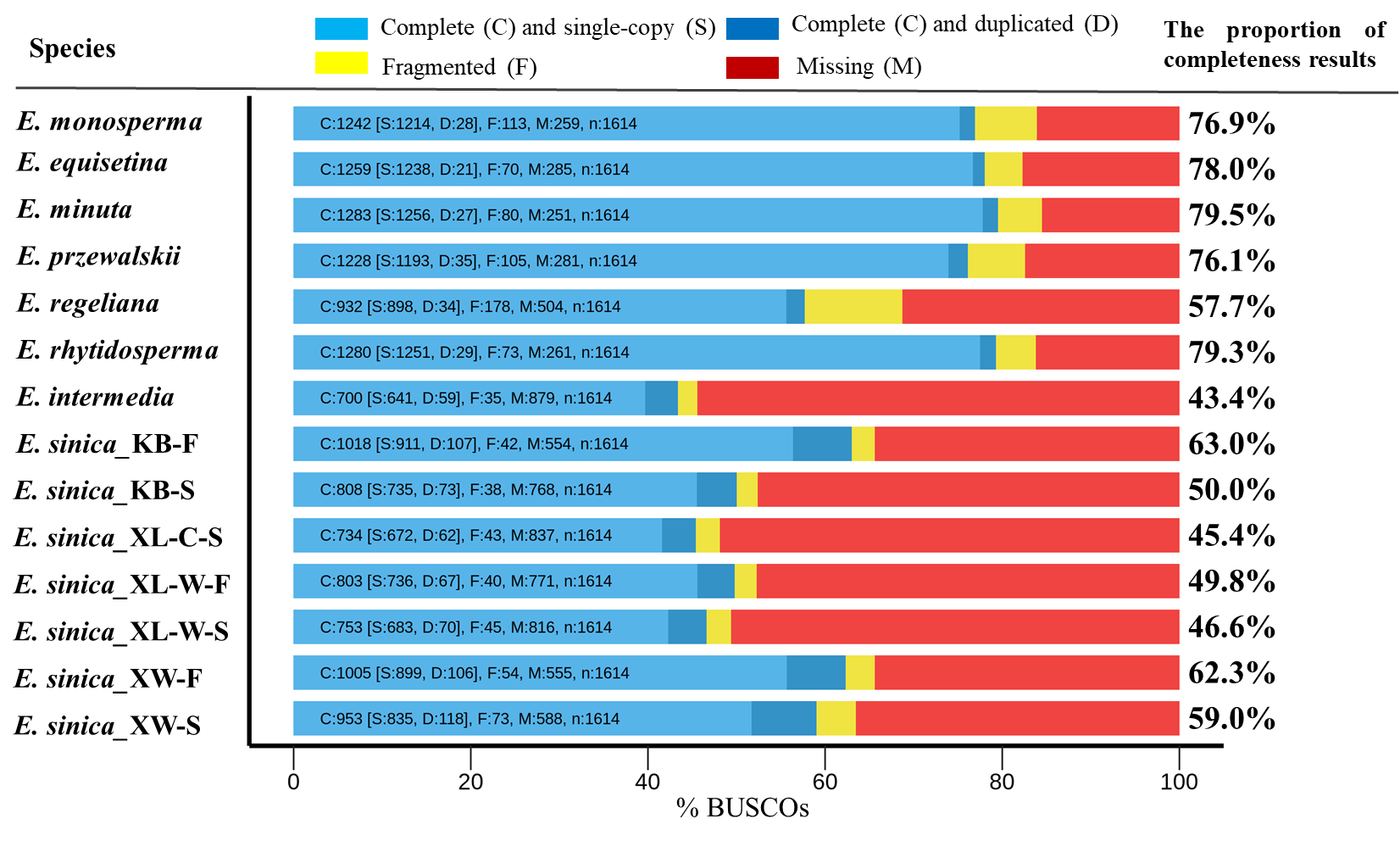
**

**Fig. S3 Results of the BUSCO assessment**


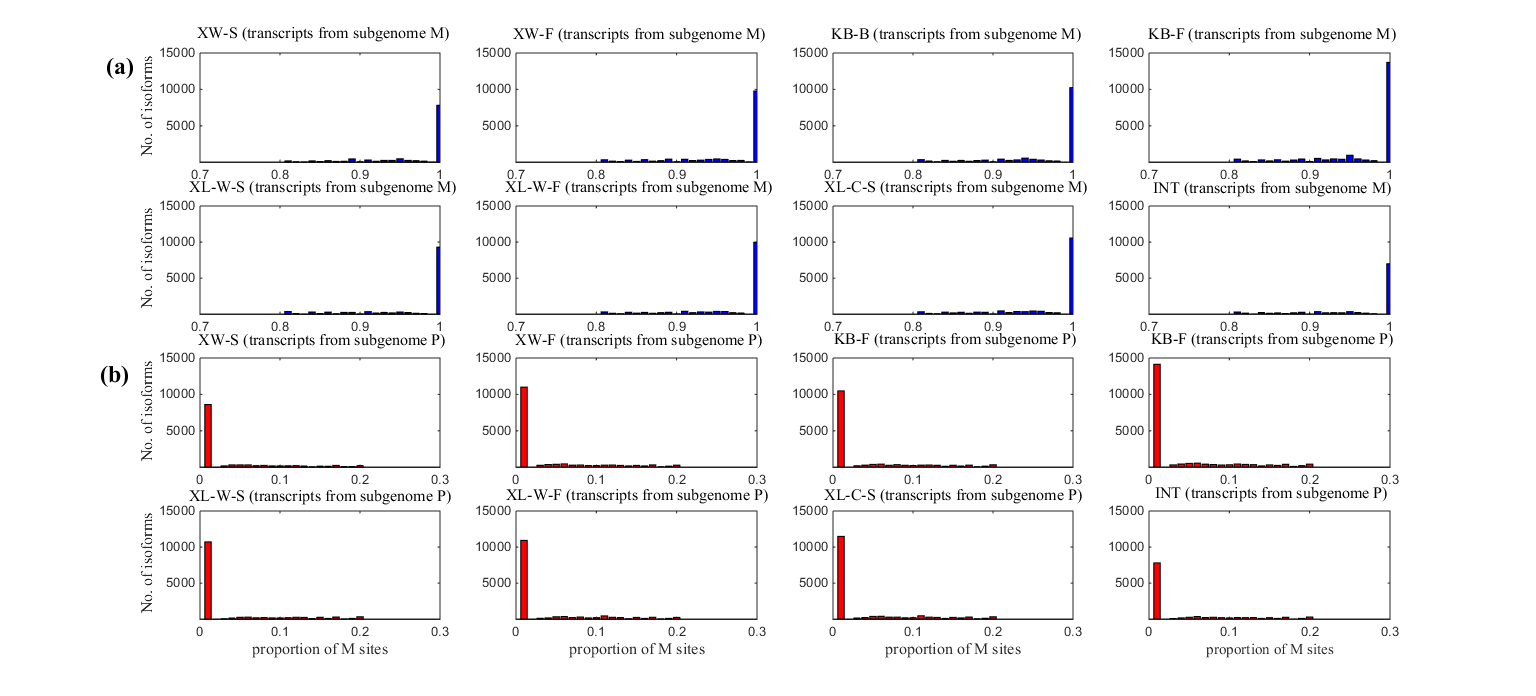


**Fig. S4 Proportion of M sites per transcript from subgenome M (a) and from subgenome P (b)**


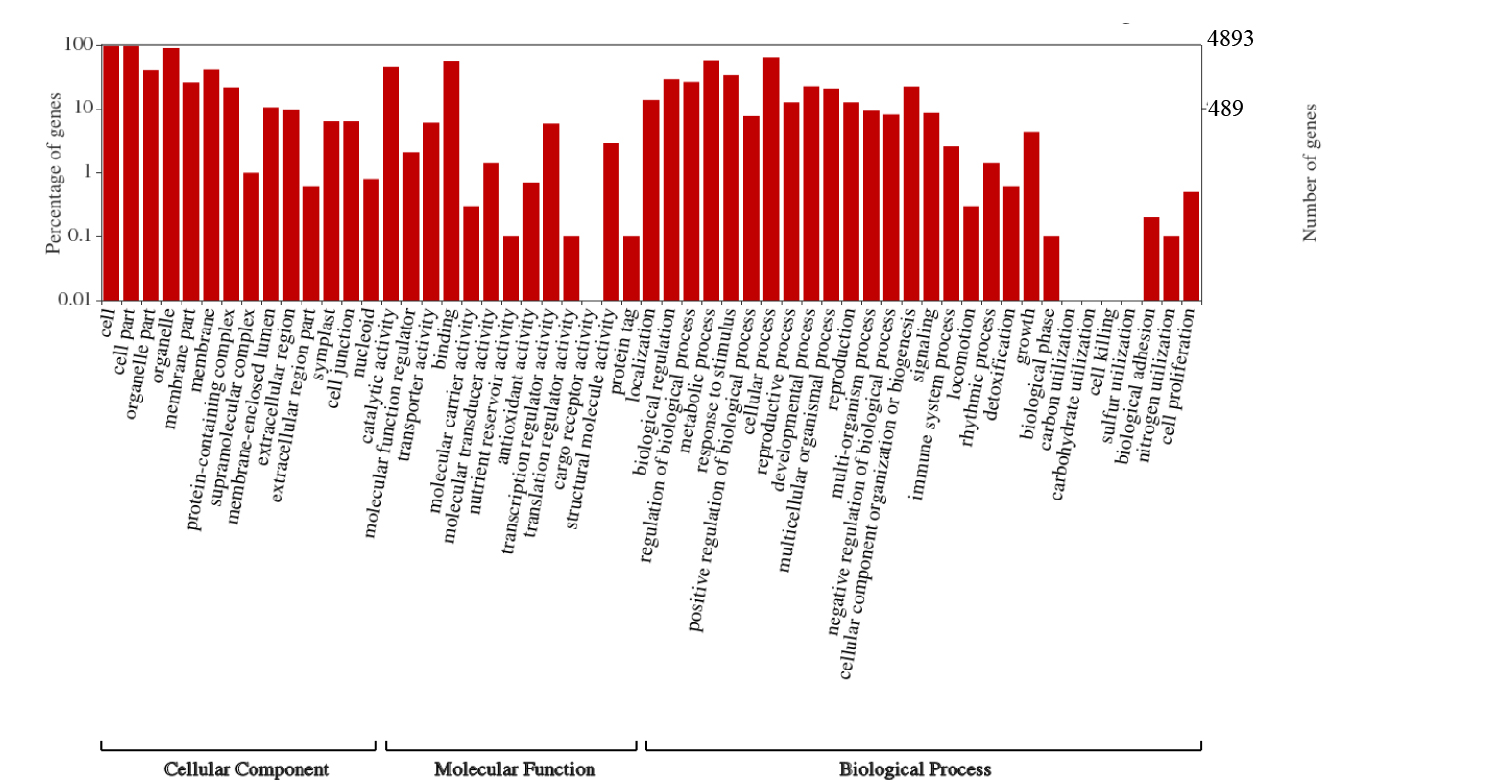


**Fig. S5 GO functional classification of the OGs. The x-axis represents the GO annotation categories and the y-axis represents the percentage of OGs of a specific category/total matched OGs**


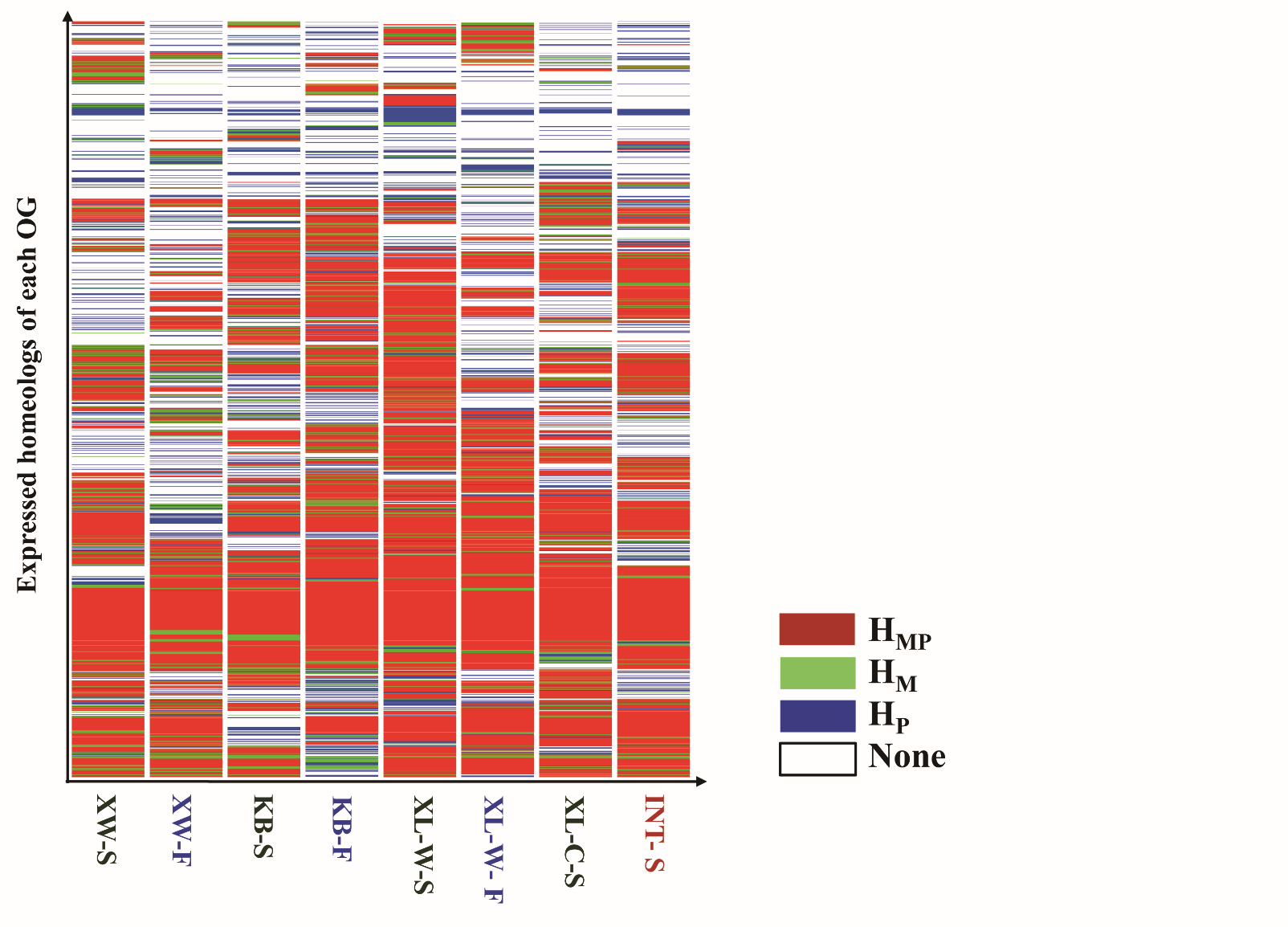


**Fig. S6 Distributions of expressed homeologs of each OG in each polyploid sample. Each row corresponds to a sample and each column corresponds to an OG**


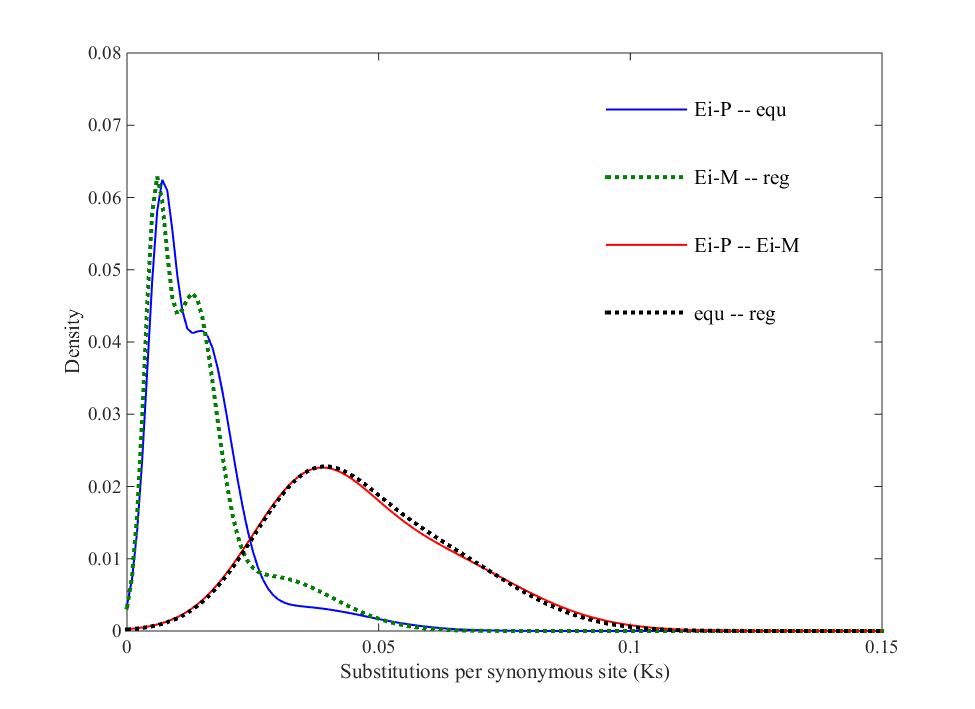


**Fig. S7 Density distribution of *Ks* among two subgenomes of *E. intermedia* and its putative diploid parents. Ei-P: subgenome P of *E. intermedia*; Ei-M: subgenome M of *E. intermedia*; equ: *E. equisetina*; reg: E. *regeliana*.**

**Table S1 Information of the transcriptome data generated from Illumina HiSeq**

| **Species** | **Ploidy**  **level** | | **Location** | **Pop.** | **Sample** | **No. of raw reads** | **No. of reads after filtering** | **Ntranscripts** | **N50 (transcripts, bp)** | **No. of**  **CDS** |
| --- | --- | --- | --- | --- | --- | --- | --- | --- | --- | --- |
| ***E. sinica*** | 4*x* | | Zhangjiakou, HB | XW | S | 22622281(100bp) | 21,987,457 |  |  |  |
|  |  |  |  |  | F | 24610161(100bp) | 23,947,858 |  |  |  |
|  |  |  | Zhangjiakou, HB | KB | S | 22210497(150bp) | 20,206,814 |  |  |  |
|  |  |  |  |  | F | 20217623(150bp) | 18,600,852 |  |  |  |
|  |  |  | Hexigten Qi, IM | XL-W | S | 20469619 (150bp) | 18,902,491 |  |  |  |
|  |  |  |  |  | F | 20503856(150bp) | 18,724,916 |  |  |  |
|  |  |  |  | XL-C | S | 22426063(150bp) | 20,526,994 |  |  |  |
| ***E. intermedia*** | 4*x* | | Shawan, XJ | INT | S | 16793661(150bp) | 15,570,339 |  |  |  |
| ***E. equisetina*** | | 2*x* | Zhangjiakou, HB | ZL | S | 32120999 (100bp) | 30,714,049 | 69343 | 1649 | 30158 |
| ***E. monosperma*** | | 2*x* | Urumqi, XJ | YX | S | 39975918 (100bp) | 37,104,728 | 41602 | 1703 | 27102 |
| ***E. minuta*** | | 2*x* | Menyuan,QH | MY | S | 30513724 (100bp) | 29,722,252 | 85304 | 1603 | 34623 |
| ***E. przewalskii*** | | 2*x* | Karamay, XJ | KLMY | S | 56506068 (100bp) | 51,820,340 | 55446 | 1553 | 28992 |
| ***E. regeliana*** | | 2*x* | Urumqi, XJ | YS | S | 33277601 (100bp) | 30,074,428 | 66873 | 1464 | 26883 |
| ***E. rhytidosperma*** | | 2*x* | Yinchuan, NX | RHY | S | 33903852 (100bp) | 32,633,573 | 69092 | 1541 | 32414 |

S: young stems; F: female strobili; W: wild; C: cultivated.

**Table S2 Information of the transcriptome data generated from PacBio Iso-Seq**

| **Sample**  **Category** | | ***E. sinica*** | | | | | | | ***E. intermedia*** |
| --- | --- | --- | --- | --- | --- | --- | --- | --- | --- |
|  |  | **XW** | | **KB** | | **XL-W** | | **XL-C** | **INT** |
|  |  | **S** | **F** | **S** | **F** | **S** | **F** | **S** | **S** |
| Clean Data | No. of polymerase bases (G) | 16.7 | 20.6 | 19.1 | 30.0 | 22.1 | 21.0 | 21.5 | 20.9 |
|  | No. of polymerase reads | 1129502 | 1035706 | 929043 | 764474 | 586803 | 566519 | 688763 | 481241 |
|  | Mean length of polymerase reads (bp) | 14752 | 19958 | 20608 | 39234 | 37666 | 37096 | 31280 | 43461 |
| CCS  (circular consensus sequence) | Mean number of passes | 12 | 14 | 20 | 31 | 43 | 32 | 28 | 37 |
| Classification | No. of full-length non-chimeric reads | 422241 | 454936 | 455372 | 504052 | 314106 | 312397 | 363854 | 262120 |
|  | Mean length of full-length non-chimeric reads (bp) | 2038 | 2276 | 1614 | 1719 | 1290 | 1653 | 1726 | 1601 |
| Cluster | No. of consensus transcripts | 179191 | 184925 | 159591 | 184929 | 123351 | 131275 | 152536 | 98103 |
|  | Mean length of consensus transcripts (bp) | 2015 | 2257 | 1671 | 1721 | 1738 | 1350 | 1785 | 1655 |
|  | No. of high-quality consensus transcripts | 34517 | 38655 | 26623 | 46439 | 26370 | 28437 | 29753 | 21950 |
|  | Mean length of high-quality consensus transcripts (bp) | 2238 | 2408 | 1665 | 1768 | 1707 | 1344 | 1735 | 1656 |

S: young stems; F: female strobili.

**Table S3 Information of analyzed transcripts**

| **Species** | **Pop.** | **Sample** | **No. of all consensus transcripts** | **No. of analyzed consensus transcripts** | **proportion** | **No. of high-quality consensus transcripts** | **No. of analyzed high-quality consensus transcripts** | **proportion** | **No. of CDS** | **No. of OGs** | **proportion** |
| --- | --- | --- | --- | --- | --- | --- | --- | --- | --- | --- | --- |
| ***E. sinica*** | XW | S | 179191 | 22017 | **12.29%** | 34517 | 9292 | **26.92%** | 19870 | 3509 | **17.66%** |
|  |  | F | 184925 | 28392 | **15.35%** | 38655 | 11338 | **29.33%** | 22300 | 3995 | **17.91%** |
|  | KB | S | 159591 | 25454 | **15.95%** | 26623 | 8122 | **30.51%** | 14858 | 3614 | **24.32%** |
|  |  | F | 184929 | 37892 | **20.49%** | 46439 | 14090 | **30.34%** | 19654 | 4389 | **22.33%** |
|  | XL-W | S | 123351 | 22445 | **18.20%** | 26370 | 8074 | **30.62%** | 13484 | 3438 | **25.50%** |
|  |  | F | 131275 | 24906 | **18.97%** | 28437 | 8556 | **30.09%** | 14412 | 3575 | **24.81%** |
|  | XL-C | S | 152536 | 27224 | **17.85%** | 29753 | 8879 | **29.84%** | 15268 | 3514 | **23.02%** |
| ***E. intermedia*** | INT | S | 98103 | 17984 | **18.33%** | 21950 | 6612 | **30.12%** | 11938 | 3097 | **25.94%** |
|  | total | | 1213901 | 206314 | **17.00%** | 252744 | 74963 | **29.66%** | 131784 | 29131 | **22.11%** |
| ***E. equisetina*** | ZL | S |  |  |  |  |  |  | 30158 | 6245 | **20.71%** |
| ***E. monosperma*** | YX | S |  |  |  |  |  |  | 27102 | 6245 | **23.04%** |
| ***E. minuta*** | MY | S |  |  |  |  |  |  | 34623 | 6245 | **18.04%** |
| ***E. przewalskii*** | KLMY | S |  |  |  |  |  |  | 28992 | 6245 | **21.54%** |
| ***E. regeliana*** | YS | S |  |  |  |  |  |  | 26883 | 6245 | **23.23%** |
| ***E. rhytidosperma*** | RHY | S |  |  |  |  |  |  | 32414 | 6245 | **19.27%** |

**Table S4 Summary of isoform distribution**

| **Samples** | | | **No. of isoforms** | **No. of isoforms per OG** | | | **OGs with 1-4 isoforms** | |
| --- | --- | --- | --- | --- | --- | --- | --- | --- |
|  |  |  |  | **Min** | **Max** | **Mean** | **H_M_ /T** | **H_P_ /T** |
| ***E. sinica*** | XW | S | 22017 | 1 | 492 | 6.3 | 497/614(81%) | 579/731(79%) |
|  |  | F | 28392 | 1 | 467 | 8.1 | 465/641(73%) | 497/718(69%) |
|  | KB | S | 25454 | 1 | 391 | 7.3 | 345/480(72%) | 382/563(68%) |
|  |  | F | 37892 | 1 | 472 | 10.8 | 325/504(64%) | 359/600(60%) |
|  | XL-W | S | 22445 | 1 | 498 | 6.4 | 354/575(62%) | 378/606(62%) |
|  |  | F | 24906 | 1 | 500 | 7.1 | 348/534(65%) | 371/560(66%) |
|  | XL-C | S | 27224 | 1 | 497 | 7.8 | 309/470(66%) | 354/552(64%) |
| ***E. intermedia*** | INT | S | 17984 | 1 | 499 | 5.1 | 414/576(72%) | 463/647(72%) |

H_M_: OGs with expressed homeologs only from subgenome M; H_P_: OGs with expressed homeologs only from subgenome P; T: total number of identified OGs; S: young stems, F: female strobili.

**Table S5 Distribution patterns of expressed homeologs based on phylogenetic approach**

| **Sample**  **Category** | ***E. sinica*** | | | | | | | | | | ***E. intermedia*** | |
| --- | --- | --- | --- | --- | --- | --- | --- | --- | --- | --- | --- | --- |
|  | **XW** | | | **KB** | | | **XL-W** | | | **XL-C** | **INT** | |
|  | **S** | **F** | **S+F** | **S** | **F** | **S+F** | **S** | **F** | **S+F** | **S** | **S** |  |
| **Total** | 3072 | 2856 | 3796 | 3158 | 3787 | 4034 | 3000 | 3132 | 3701 | 3085 | 2777 |  |
| **H_MP_** | 2253  (73%) | 2126（74%） | 3175  (84%) | 2498  （79%） | 3121（82%） | 3676  (91%) | 2257  (75%) | 2397（76%） | 3334  (90%) | 2435  (79%) | 1948  (70%) |  |
| **H_M_** | 453  (15%) | 398（14%） | 342  (9%) | 371  (12%) | 370  (10%) | 203  (5%) | 417  (14%) | 428  (14%) | 194  (5%) | 340  (11%) | 458  (16%) |  |
| **H_P_** | 366  (12%) | 332（12%） | 279  (7%) | 289  (9%) | 296  (8%) | 155  (4%) | 326  (11%) | 307  (10%) | 173  (5%) | 310  (10%) | 371  (14%) |  |

H_MP_: OGs with expressed homeolog pairs from both subgenomes; H_M_: OGs with expressed homeologs only from subgenome M; H_P_: OGs with expressed homeologs only from subgenome P; S: young stems, F: female strobili.

**Table S6 Pearson’s correlation coefficients between polyploid samples based on expression patterns of homeologs**

| **Sample** | | ***E. sinica*** | | | | | | | ***E. intermedia*** |
| --- | --- | --- | --- | --- | --- | --- | --- | --- | --- |
|  |  | **Xw** | | **Kb** | | **Xl-w** | | **Xl-C** | **Int** |
|  |  | **S** | **f** | **S** | **f** | **S** | **f** | **S** | **S** |
| **Xw** | **S** | 1 | 0.40 | 0.26 | 0.29 | 0.20 | 0.29 | 0.33 | 0.25 |
|  | **f** | 0.40 | 1 | 0.30 | 0.36 | 0.23 | 0.33 | 0.38 | 0.29 |
| **Kb** | **S** | 0.26 | 0.30 | 1 | 0.37 | 0.30 | 0.37 | 0.37 | 0.34 |
|  | **f** | 0.29 | 0.36 | 0.37 | 1 | 0.25 | 0.35 | 0.35 | 0.31 |
| **Xl-w** | **S** | 0.20 | 0.23 | 0.30 | 0.25 | 1 | 0.35 | 0.29 | 0.32 |
|  | **f** | 0.29 | 0.33 | 0.37 | 0.35 | 0.35 | 1 | 0.42 | 0.34 |
| **Xl-C** | **S** | 0.33 | 0.38 | 0.37 | 0.35 | 0.29 | 0.42 | 1 | 0.37 |
| **Int** | **S** | 0.25 | 0.29 | 0.34 | 0.31 | 0.32 | 0.34 1 | 0.37 | 1 |

**Table S7 Complete mixture model estimates of *Ks* distributions**

|  | G (#Gaussian components) | Mean *Ks* | variance | Fraction of data | Merged peak | estimated divergence times (Mya) |
| --- | --- | --- | --- | --- | --- | --- |
| Es-P--equ | 4 | 0.0109 | 0.0035 | 0.4215 | 0.0110 | 2.29 |
|  |  | 0.0353 | 0.0143 | 0.0726 |  |  |
|  |  | 0.0193 | 0.0063 | 0.2676 |  |  |
|  |  | 0.0052 | 0.0017 | 0.2384 | 0.0060 | 1.25 |
| Es-M--reg | 4 | 0.0050 | 0.0017 | 0.2065 | 0.0050 | 1.04 |
|  |  | 0.0350 | 0.0139 | 0.0838 |  |  |
|  |  | 0.0108 | 0.0036 | 0.3823 | 0.0110 | 2.29 |
|  |  | 0.0201 | 0.0067 | 0.3274 |  |  |
| Es-P--Es-M | 2 | 0.0597 | 0.0192 | 0.4196 |  |  |
|  |  | 0.0377 | 0.0122 | 0.5804 | 0.0400 | 8.33 |
| Ei-P--equ | 3 | 0.0066 | 0.0027 | 0.3226 | 0.0070 | 1.46 |
|  |  | 0.0333 | 0.0141 | 0.1181 |  |  |
|  |  | 0.0150 | 0.0056 | 0.5594 | 0.0150 | 3.13 |
| Ei-M--reg | 3 | 0.0056 | 0.0022 | 0.2607 | 0.0060 | 1.25 |
|  |  | 0.0283 | 0.0126 | 0.2362 |  |  |
|  |  | 0.0129 | 0.0470 | 0.5030 | 0.0130 | 2.71 |
| Ei-P--Ei-M | 2 | 0.0581 | 0.0184 | 0.4971 | 0.0390 | 8.13 |
|  |  | 0.0358 | 0.0118 | 0.5029 |  |  |
| equ--reg | 2 | 0.0358 | 0.0117 | 0.4813 | 0.0390 | 8.13 |
|  |  | 0.0569 | 0.0174 | 0.5187 |  |  |

Es-P: subgenome P of *E. sinica*; Es-M: subgenome M of *E. sinica*; Ei-P: subgenome P of *E. intermedia*; Ei-M: subgenome M of *E. intermedia*; equ: *E. equisetina*; reg: E. *regeliana*.
